# Screening of Peruvian Medicinal Plants for Tyrosinase Inhibitory Properties: Identification of Tyrosinase Inhibitors in *Hypericum laricifolium* Juss.

**DOI:** 10.3390/molecules22030402

**Published:** 2017-03-04

**Authors:** Yanymee Nimesia Guillen Quispe, Seung Hwan Hwang, Zhiqiang Wang, Soon Sung Lim

**Affiliations:** 1 Department of Food Science and Nutrition, Hallym University, 1 Hallymdeahak-gil, Chuncheon 24252, Korea; estreyany@gmail.com (Y.N.G.Q.); isohsh@gmail.com (S.H.H.); wangzq01234@gmail.com (Z.W.); 2 Institute of Natural Medicine, Hallym University, 1 Hallymdeahak-gil, Chuncheon 24252, Korea; 3 Institute of Korean Nutrition, Hallym University, 1 Hallymdeahak-gil, Chuncheon 24252, Korea

**Keywords:** Peruvian plants, tyrosinase, screening, *Hypericum laricifolium* Juss.

## Abstract

Tyrosinase inhibitors are of far-ranging importance in cosmetics, medicinal products, and food industries. Peru is a diverse country with a wide variety of plants that may contain excellent anti-tyrosinase inhibitors. In the present study, the tyrosinase inhibitory properties of 50 medicinal plant extracts from Peru were investigated using tyrosinase assay. Among plant extracts, those that showed an inhibition rate >50% were *Hypericum laricifolium* Juss., *Taraxacum officinale* F.H.Wigg., and *Muehlenbeckia vulcanica* Meisn., with *H. laricifolium* Juss. showing the greatest anti-tyrosinase activity. Although *H. laricifolium* Juss. has been widely used as a medicinal plant by Peruvians, little is known regarding its bioactive components and effects on tyrosinase activity. For this reason, we attempted to discover tyrosinase inhibitors in *H. laricifolium* Juss. for the first time. The bioactive components were separated by Sephadex LH-20 chromatography and eluted with 100% methanol. Eight compounds were discovered and characterized by high-performance liquid chromatography coupled with diode array detection (HPLC-DAD): protocatechuic acid, *p*-hydroxybenzoic acid, chlorogenic acid, vanilic acid, caffeic acid, kaempferol 3-*O*-glucuronide, quercetin, and kaempferol. In addition, the concentration of these compounds required for 50% inhibition (IC_50_) of tyrosinase activity were evaluated. Quercetin exhibited the strongest tyrosinase inhibition (IC_50_ 14.29 ± 0.3 μM). Therefore, the Peruvian plant *H. laricifolium* Juss. could be a novel source for anti-tyrosinase activity.

## 1. Introduction

Tyrosinase (E.C.1.14.18.1), also known as polyphenol oxidase, is a multifunctional glycosylated copper-containing enzyme which is widespread in many organisms including animals, plants, and microorganisms [1]. Tyrosinase is mainly involved in the initial steps of the pathway which catalyzes the orthohydroxylation of l-tyrosine to l-3,4-dihydroxyphenylalanine (l-DOPA) (monophenolase activity) and the oxidation of l-DOPA to dopaquinone (diphenolase activity), which is then converted to the end-product melanin [2,3]. Melanin is the main component responsible for the darkening of the skin and hair, and plays an important role against ultraviolet (UV) ray damage. However, the accumulation of an excessive level of melanin can cause skin damage, such as age spots or malignant melanoma. It has also been associated with Parkinson’s disease [4]. Additionally, the browning of fruits and vegetables is related to the oxidation of phenolic compounds catalyzed by tyrosinase, which results in a loss of market value for the food [2,3,4,5]. Therefore, tyrosinase inhibitors are important in cosmetics (hyperpigmentation), medicinal products, and food industries. To date, despite the existence of a large number of tyrosinase inhibitors, only a few are marketed as safe [3,6]. Thus, the search for new tyrosinase inhibitors is important for the treatment of hyperpigmentation, development of skin-whitening agents, and use as preservatives in the food industry.

Peru is a developing country characterized by a rich biodiversity, where medicinal plants still represent the main therapeutic tool in traditional medicine, especially in many ethnic groups. It is estimated that 17,144 flowering plant species exist in Peru, of which 5354 (31.3%) are endemic, while the rest are native or introduced [7,8]. Some of these may be good candidates for the development of new anti-tyrosinase agents and/or standardized phytomedicines, and may be effective as future therapies for various diseases. However, to date, no report has been published regarding the tyrosinase inhibitory activity of Peruvian plants.

For that reason, as part of our ongoing efforts to find new tyrosinase agents, we measured the anti-tyrosinase activity of 50 Peruvian plants traditionally used as medicinal infusions by Peruvian people, based on the ethnobotanical information provided by literature sources [9,10,11,12,13,14,15,16,17]. Among the extracts that were screened, we selected the most active plants against tyrosinase based on spectrophotometric analyses of their bioactive compounds. Subsequently, we found that *Hypericum laricifolium* Juss. (A10) extract showed significant tyrosinase inhibition. *H. laricifolium* Juss., is a species of *Hypericum* (Clusiaceae) which can be found in tropical regions of the world at high altitudes, particularly in South America and Africa. In South America, *H. laricifolium* Juss., is distributed from western Venezuela, along the Andes of Colombia and Ecuador through central Peru and into Bolivia. In Peru it is called “Hierba de la fortuna” and used for good health, fortune, or luck in love, while in Ecuador it is called “Romerillo” or “Hierba de San juan” and used as a diuretic and for inducing menstruation [18,19].

In one study, from the aerial parts of *H. laricifolium Juss.,* twelve xanthones were isolated and identified [20]. Biological investigations also revealed the presence of phenolic acids, flavonoids, triterponoids [21], and dimeric and monomeric acylphloroglucinol derivatives [22,23,24]. Additionally, studies in Venezuela reported the chemical composition of *H. laricifolium* Juss. essential oils [25]. Scientists in Peru also found that the leaves of *H. laricifolium* Juss. had an anti-depressive effect in rats [22]. To the best of our knowledge, this plant has not previously been studied relative to its effect on tyrosinase. In this study, for the first time, we investigated inhibitory effects and characterization of compounds with an effect on tyrosinase extracted from *H. laricifolium* Juss.

## 2. Results and Discussion

### 2.1. Ethnopharmacological Data

Plants represent a rich source of bioactive chemicals, many of which are largely free from harmful adverse effects [26,27], but their individual activity is not sufficiently potent to be of practical use. Recently, safe and effective tyrosinase inhibitors have become important for their potential applications in improving the quality of food, preventing pigmentation disorders, and preventing other melanin-related health problems in human beings, in addition to cosmetic applications [28]. We selected 50 species to be studied which were bought in popular markets in Lima, Peru. These plants were chosen due to the fact that there have been no previous studies on their effect on tyrosinase activity. Table 1 provides the scientific name, the common name, the family name, as well as the traditional use of each plant. Many of the plants had two or more names which could be found in Spanish or in the native language Quechua. All the plants chosen are used by Peruvians, and have different traditional uses, such as for treatment of liver diseases, use as an anti-inflammatory, or for curing cancer, diabetes, and other diseases. These 50 species belong to 29 botanical different families. Asteraceae was the family with the largest number medicinal species (18%), followed by the Fabaceae, Lamiaceae, and Euphorbiacea families (10%, 8%, 8%).

### 2.2. Screening of Tyrosinase Inhibitory Activities of Methanol Extracts

Crude extracts of 50 plants were prepared using 70% methanol and their yields were from 1.1% to 57.1% as listed in Table 2.

For the extraction of the 50 plants, an assortment of plant parts were used in our study, including leaves, flowers, aerial parts, seeds and roots, according to the traditional use by Peruvians. The effect on tyrosinase inhibition of the 50 crude methanol extracts were investigated at a concentration of 500 μg/mL and the results are reported in Table 2. Arbutin was used as the positive control. Of the 50 extracts assayed, 36 extracts demonstrated an effect on tyrosinase activity, among which three showed an inhibition rate >50%, 5 showed inhibition rates between 40% and 50%, and the rest showed in inhibition rate <40%. The best inhibitory activities were observed in extracts of leaves of *H. laricifolium* Juss. which showed 74% ± 2.1% inhibition, followed by *Taraxacum officinale* F.H. Wigg (P49, inhibition percent is 60.8% ± 4.1%), *Muehlenbeckia vulcanica* Meisn (P82, inhibition percent is 57.1% ± 3.0%), *Salvia hispanica* L. (P4, inhibition percent is 44.3% ± 3.4%), *Senna* sp. (A7, inhibition percent is 44.1% ± 2.3%), *Anacardium occidentale* L. (A41, inhibition percent is 42.5% ± 7.2%), *Matricaria recutita* L. (A12, inhibition percent is 40.49% ± 2.1%) and *Ch. pilosa* Goldm (P17, inhibition percent is 40.35% ± 2.0%).

In Korea, one study reported on the antioxidant capacity and tyrosinase inhibition activity of water extracts of various parts of *T. Officinale.* The leaf extract (1 mg/mL) showed the highest tyrosinase inhibition (34.2%), while the roots and whole plant showed tyrosinase inhibition of more than 20% [32]. *M. volcanica* contains anthraquinone-*O*-glycosides, and according to some investigations, some anthraquinones have an anti-tyrosinase effect [33,34]. *S. hispanica* L. contains compounds such as quercetin, kaemperol and caffeic acids, and these are known to have anti-tyrosinase activity [35]. *A. occidentale* L. was found to exhibit anti-tyrosinase activity [36], while *M. recutita* L. showed an ability to reduce ultraviolet B (UVB)–induced pigmentation in vivo [37]. There are no reports on anti-tyrosinase activity in the other samples.

**Figure 1:**
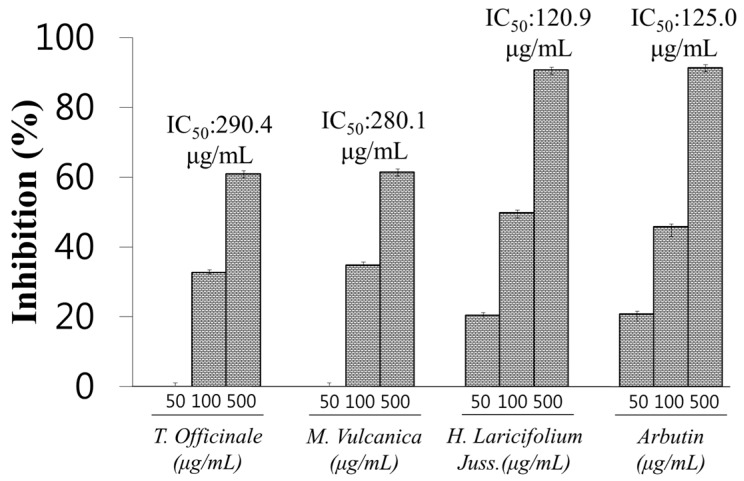
Tyrosinase inhibitory effect of the most effective extracts of Peruvian medicinal plants, *T. Officinale* F.H. Wigg, *M.Vulcanica* Meisn, *H. Laricifolium Juss.* Arbutin was used as positive control. IC_50_, indicates the concentration of each extract required for 50% inhibition of the enzyme activity.

As Figure 1 shows, the extracts which showed an inhibition rate >50%, were selected and their IC_50_ (concentration of each extract required for 50% inhibition of the enzyme activity) was determined with *H. laricifolium* Juss., *T. officinale* F.H. Wigg, and *M. vulcanica* Meisn, with an IC_50_ of 120.9 μg/mL, 280.1 μg/mL, and 290.4 μg/mL, respectively. Because of its low IC_50_ and due to its high availability, *H. laricifolium* Juss. was selected as a potential source of a new tyrosinase inhibitor.

### 2.3. Effect of H. laricifolium *Juss.* on Tyrosinase Inhibition

*H. laricifolium Juss.* has many traditional uses. In Peru, it is distributed in Amazonas, Ancash, Cajamarca, Huanuco, La Libertad, Pasco, Piura, and San Martin, between 2000 and 4500 m above sea level [17]. Previous studies have reported that *H. laricifolium* Juss. has various phytochemical constituents, including two caffeic acid esters of long-chain aliphatic alcohols, sterols, triterpenoids, benzoic and cinnamic acid derivatives [21,22,23,24,25], flavonols, and flavonol glycosides. These components were tested in vitro for anti-inflammatory activity, especially quercetin, which inhibited cyclooxygenase-1 (COX-1) by 52% ± 2% and caffeic acid esters which inhibited COX-2 by 44% ± 2% [21].

In other studies, the ethanol extracts of leaves of *H. laricifolium* Juss. showed a weak minimum inhibitory concentration (MIC) of 250 μg/mL against *Candida albicans*, but the bioactive compounds were not identified [38]. In the literature, no information has been found regarding tyrosinase activity in *H. laricifolium* Juss., but there are reports on one species of *Hypericum* of which St. John’s Wort (*Hypericum perforatum* L.) is the most well-known. It is a medicinal herb with antidepressant activity and is possibly associated with anti-cholinesterase, anti-tyrosinase, and antioxidant properties [20,39].

Its known that genus *Hypericum* has a variety of molecules with different biological activities, among them the content of hypericin and various phenolics is considerable, exhibiting wide pharmacological activities such as anti-inflammatory, anti-cholinesterase, antimicrobial, antiviral and antioxidant properties [40]. The species *Hypericum humifusum* and *Hypericum perfoliatum* have great content of hypericin and phloglucinols. Hypericin is used against viruses and retroviruses such as the human immunodeficiency virus (HIV) 35, and influenza A, while phloroglucinols (hyperforin and adhyperforin) show potential antibacterial, antidiabetic, and cytotoxic activities [40,41].

According to our results mentioned above, we decided to compare the differences in anti-tyrosinase activities between 70% and 50% methanol extracts, and a methylene chloride extract of *H. laricifolium* Juss. at concentrations of 100, 500, and 1000 μg/mL, and arbutin. As shown in Table 3, we found that the methylene chloride extract did not show any activity and the 50% methanol extract showed low inhibition at 1000 μg/mL (22.3%). The 70% methanol extract demonstrated a good inhibition of tyrosinase activity; the inhibition rate was >50% at 500, and 1000 μg/mL (IC_50_ is 122.1 μg/mL). The IC_50_ value for arbutin was 42.0 μg/mL. According these results, a 70% methanol extract should be studied to determine the constituents responsible for tyrosinase inhibition.

### 2.4. Identification of Major Bioactive Components of H. laricifolium *Juss.*

**Figure 2:**
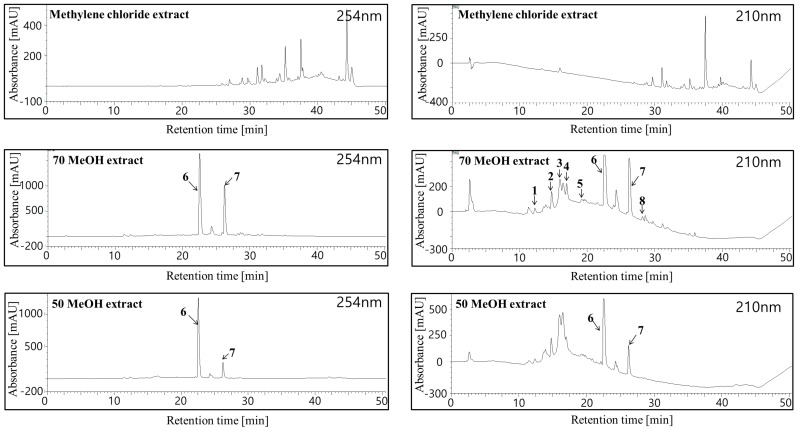
HPLC chromatograms of crude *H. laricifolium* Juss. methylene chloride extract, 70% methanol extract, and 50% methanol extract at 254 nm and 210 nm.

**Figure 3:**
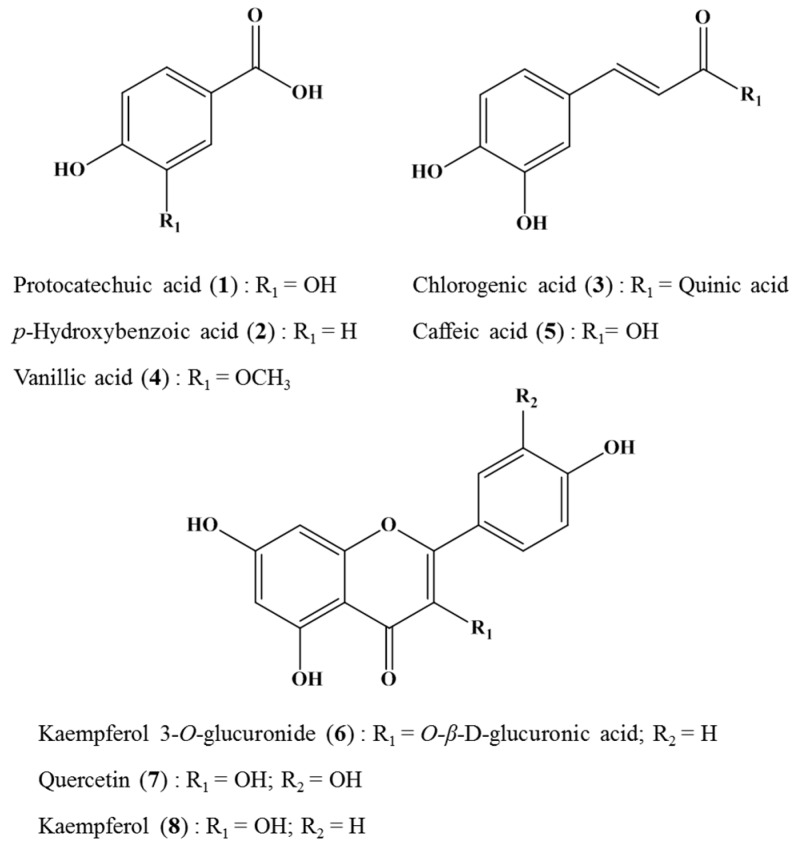
Compounds isolated from crude *H. laricifolium* Juss. extract by Sephadex LH-20 chromatography.

Figure 2 shows the HPLC chromatograms of crude *H. laricifolium* Juss. extracts. All the sample components were separated in less than 40 minutes, with retention factors depending mainly on structural hydrophobicity, as previously demonstrated by a number of studies employing the HPLC for highly efficient separations of complex samples [42]. After the isolation, we identified and isolated eight compounds as candidates for tyrosinase inhibition, eluting in different retention times. From compound (**1**) to compound (**5**), peaks appeared between 12 and 19 min, and from compound (**6**) to compound (**8**), peaks appeared between 20 and 30 min. Figure 3 shows the chemical structures of these isolated compounds. From the 70% methanol extract of the plant, protocatechuic acid was obtained and identified as compound (**1**), *p*-hydroxybenzoic acid as compound (**2**), chlorogenic acid as compound (**3**), vanillic acid as compound (**4**), caffeic acid as compound (**5**), kaempferol 3-*O*-glucuronide as compound (**6**), quercetin as compound (**7**) and kaempferol compound as (**8**). These compounds were positively identified both on the basis of information provided by the literature, by direct comparisons with authentic materials available commercially, and by comparison with data from similar species in the same family [21,43,44].

### 2.5. Effect of Bioactive Compounds on Tyrosinase Inhibition

The tyrosinase inhibitory activities of the major bioactive components of *H. laricifolium* Juss. were confirmed using a tyrosinase assay. As shown in Table 4, all compounds except quercetin (compound **7**), did not show any anti-tyrosinase activity. Quercetin showed strong tyrosinase inhibition with an IC_50_ of 14.29 ± 0.3 μM, which is seven times lower than that of arbutin (IC_50_ = 110.4 ± 1.9 μM) but a little higher than that of kojic acid (IC_50_ 8.0 ± 0.5 μM), which is also a positive control produced by many species of fungi such as *Aspergillus* and *Penicillium* sp. [27]. Although some other compounds showed some tyrosinase inhibitory activities at 500 μg/mL or 1000 μg/mL (data not shown), there were no other strong inhibitors.

The inhibition percent of original methanol extract of *H. laricifolium* Juss. (data not shown) and quercetin at 100 µg/mL of concentration were compared, the methanol extract show around 50% inhibition, while quercetin show high inhibition over 95%. Therefore, it is revealed that quercetin has a significant inhibition on tyrosinase activity compared to the other compounds and is the main determinant of the observed activity for *H. laricifolium* Juss. The x-axis and y-axis is fuzzy.

In previous studies on flavonoids, compounds such as quercetin and kaempferol were shown to interfere with the activity of tyrosinase through chelation of copper in its active site [28]. It has been demonstrated that quercetin and kaempferol can suppress melanogenesis, directly inhibiting tyrosinase activity or reducing tyrosinase expression [45]. In our study, quercetin proved to be a good tyrosinase inhibitor, while kaempferol was a weaker inhibitor than arbutin.

**Figure 4:**
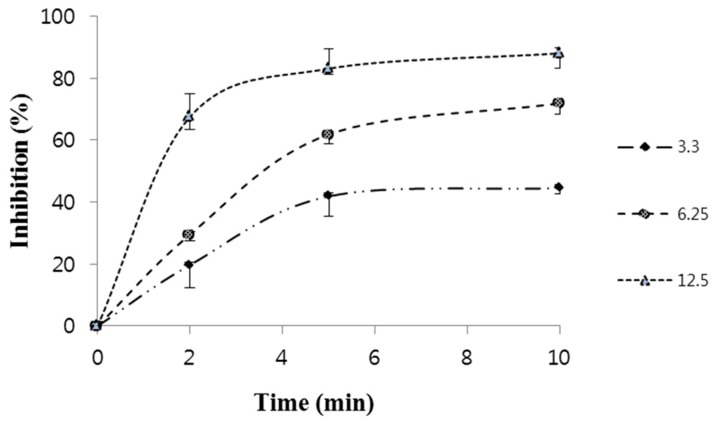
Course of oxidation of L-tyrosine by tyrosinase in the presence of different concentrations of quercetin (compound **7**). Concentrations were 3.3, 6.25, and 12.5 μg/mL.

Figure 4 shows % inhibition of quercetin using L-tyrosine as a substrate, we decided to study quercetin’s ability to inhibit the activity of tyrosinase at different times and in different concentrations (2 min, 5 min, 10 min, and 3 μg/mL, 6.25 μg/mL, and 12.5 μg/mL). At a concentration of 12.5 μg/mL, quercetin inhibited >50% tyrosinase at 2 min, 5 min, and 10 min, with the greatest inhibition being 88.1%.

As quercetin is the only active compound in *H. laricifolium Juss.* responsible for tyrosinase inhibition, the 70% methanol extract, with the highest concentration of quercetin, showed the strongest activity against tyrosinase, more than the 50% methanol extract and methylene chloride extract. This study is a starting point for further investigations on tyrosinase inhibitors with respect to Peruvian plant extracts. Simultaneously, we suggest that more Peruvian plants should be explored in order to identify more useful chemical compounds.

## 3. Materials and Methods

### 3.1. Ethnobotanical Search

The plants were selected according to the ethnobotanical bibliography provided by different literature sources [9,10,11,12,13,14,15,16,17,29,30,31], particularly those with a lack of scientific information about their activity. Scientific names, common names which can be found in Spanish or Quechua (native language in Peru), and traditional uses for Peruvian population are summarized in Table 1.

### 3.2. Chemicals

Mushroom tyrosinase (120 kDa), l-tyrosine, propylene glycol, dimethyl sulfoxide, arbutin, and kojic acid were obtained from Sigma-Aldrich Chemical Co. (St. Louis, MO, USA). Potassium phosphate dibasic (K_2_HPO_4_), and potassium phosphate monobasic (KH_2_PO_4_), were obtained from Junsei Chemical Co. (Tokyo, Japan). Deionized distilled water used for all solutions, dilutions, and HPLC analysis was obtained from a Milli-Q system (Millipore, Bedford, MA, USA) with a resistance of over 18.2 MΩ cm. The Sephadex LH-20 column was purchased from GE Healthcare (Uppasala, Sweden). All organic solvents were HPLC grade and were obtained from J.T. Baker (Phillipsburg, NJ, USA). All other chemicals and solvents, unless otherwise specified, were guaranteed reagent grade and purchased from Sigma-Aldrich Chemical Co. (St. Louis, MO, USA).

### 3.3. Plant Materials

The 50 dried plant samples were purchased from different local markets in Lima, Peru from May to October 2015. The vouchers of all samples were deposited at the Center for Efficacy Assessment and Development of Functional Foods and Drugs, Hallym University (Chuncheon, South Korea). The samples were identified according to macroscopic characters using available literature in English and Spanish and verified by Paul H. Gonzales Arce from the Museo de Historia Natural, Universidad Nacional Mayor de San Marcos, Lima, Peru.

### 3.4. Preparation of Extracts and Isolation of Plant Samples

The dried Peruvian plants (20–50 g) were pulverized at room temperature and then were extracted by maceration at room temperature with 70% methanol for 72 h. The supernatants were filtered through filter paper (Hyundai Micro, Seoul, Korea) and evaporated under vacuum at 37 °C by means of a rotary evaporator (Eyela, Tokyo Rikakikai CO, Tokyo, Japan). The resultant extract was stored at −4 °C until use. Fifty extracts were prepared, and the extractions were performed in duplicate. A yield for each extract was obtained after the solvent was removed and expressed as the calculated weight of air-dried crushed plant material with respect to the starting material.

The major components of the *H. laricifolium* Juss. 70% methanol extract were isolated by column chromatography. Briefly, the 70% methanol extract of *H. laricifolium* Juss. (1 g), was dissolved in methanol and loaded onto a Sephadex LH-20 column (2 cm × 90 cm, Uppsala, Sweden). The column was eluted with 100% methanol at a flow rate of 2.0 mL/min. The effluents were collected (fraction size 40 mL) into test tubes as 10 separate fractions. Among them, from fractions 7 and 10 the compounds **6** and **7** were directly obtained, which showed high purities: kaempferol 3-*O*-glucuronide (40.3 mg, 98.73% purity) and quercetin (16.8 mg, 98.36% purity), respectively. Six other major compounds were identified by direct comparison with the authentic materials available commercially or in our laboratory, and also by comparison with references of similar species of same family [21,41,42]. Among them were protocatechuic acid (**1**), *p*-hydroxybenzoic acid (**2**), chlorogenic acid (**3**), vanillic acid (**4**), caffeic acid (**5**) and kaempferol (**8**).

### 3.5. HPLC Analysis

HPLC analysis was carried out on a Dionex system (Dionex, Sunnyvale, CA, USA) consisting of a P850 pump, an ASI-100 automated sample injector, a Synergi Hydro-RP 80A column (150 mm × 4.6 mm, 4 μm; Phenomenex, Torrance, CA, USA) maintained at 30 °C, and an UVD170S detector. Briefly, the mobile phase was composed of 0.1% trifluoroacetic acid (A-line) and methanol (B-line). The gradient elution system was modified as follows: 0–35 min, starting with 5% B-line, programmed to reach 40% B-line at 15 min using a linear gradient, followed by 100% B-line from 15–35 min. The flow rate was 0.7 mL/min and 10 μL was the sample injection volume. The detector monitored the eluent at wavelength 254 nm.

### 3.6. NMR Analysis

Two compounds were separated from the 70% MeOH extract of *H. laricifolium* Juss. by Sephadex LH-20 column chromatography. Kaempferol 3-*O*-glucuronide (compound **6**) and quercetin (compound **7**) were identified by comparing ^1^H-NMR and ^13^C-NMR spectra with previously reported data. ^1^H-NMR spectra of these two isolated, pure compounds were recorded with a Bruker AV600 instrument (Mundelein, IL. USA), using DMSO-d_6_ as a solvent. The detailed structural information was listed as following:

***Compound 6.*** ^1^H-NMR (DMSO-*d*_6_, 600 MHz) δ 6.20 (1H, *d*, *J* = 2.1 Hz, H-6), δ 6.42 (1H, *d*, *J* = 2.0 Hz, H-8), δ 6.86 (2H, *d*, *J* = 8.9 Hz, H-3’, 5’), δ 8.00 (2H, *d*, *J* = 8.9 Hz, H-2’, 6’), δ 5.47 (1H, *d*, *J* = 7.5 Hz, H-1”), δ 3.23–3.18 (1H, m, H-2”), δ 3.53 (1H, *d*, *J* = 9.6 Hz, H-5”), δ 3.26–3.22 (1H, m, H-3”), δ 3.35–3.30 (1H, m, H-4”); p^13^ C NMR (DMSO-*d*_6_, 150 MHz) δ 157.4 (C-2), 134.3 (C-3), 177.8 (C-4), 161.0 (C-5), 99.8 (C-6), 165.5 (C-7), 94.5 (C-8), 156.8 (C-9), 103.9 (C-10), 120.6 (C-1’),160.1 (C-4’), 102.8 (C-1”), 72.0 (C-2”), 73.6 (C-3”), 76.3 (C-4”), 76.7 (C-5”), 173.8 (C-6”). The ^1^H-NMR, ^13^C-NMR data for compound **6** are identical to those reported previously [46]. Compound **6** was identified as kaempferol 3-*O*-glucuronide.

***Compound 7.*** ^1^H-NMR (DMSO-*d*_6_, 600 MHz) δ 6.19 (1H, *d*, *J* = 2.0 Hz, H-6), δ 6.40 (1H, *d*, *J* = 2 Hz, H-8), δ 6.88 (1H, *d*, *J* = 8.4 Hz, H-5’), δ 7.54 (1H, *dd*, *J* = 2.2, 8.4 Hz, H-6’), δ 7.68 (1H, *d*, *J* = 2.2 Hz, H-2’), δ 12.50 (1H, s, 5-OH). p^13^C NMR (DMSO-d_6_, 150 MHz) δ 177.3 (C-4), 165.7 (C-7), 162.5 (C-3), 158.3 (C-9), 148.1 (C-4’), 147.9 (C-2), 146.3 (C-3’), 137.3 (C-3), 124.2 (C-1’), 121.7 (C-6’), 116.3 (C-5’), 116.0 (C-2’), 104.6 (C-10), 99.3 (C-6), 94.4 (C-8). The ^1^H-NMR, ^13^C-NMAR data for compound **7** are identical to those reported previously [47,48]. Compound **7** was identified as quercetin.

### 3.7. Tyrosinase Assay

Tyrosinase activity was determined by spectrophotometry, with minor modifications [26]. First, 80 μL of 0.1 M potassium phosphate buffer (pH 6.6), 20 μL of sample dissolved in dimethyl sulfoxide at the concentrations needed (final concentrations 100–500 μg/mL), and 50 μL of l-tyrosine buffer (1.5 mM) solutions were mixed. Then, 50 μL of mushroom tyrosinase solution (200 unit/mL in phosphate buffer) was added to each mixture, which was then incubated at 25 °C for 15 min. The absorbance of the mixture was observed at 450 nm using a 96-well reader and an EL-800 Universal microplate reader (Bio-Tek Instrument Inc. Winooski, VT, USA). Arbutin and kojic acid were used as positive controls. All the samples were first tested at 500 μg/mL and those showing 50% inhibition or higher in three different repetitions were further evaluated for the concentration necessary for 50% inhibition (IC_50_). The percent inhibition of tyrosinase was calculated as follows:

(1) $$\%\;\mathrm{Inhibition}\;\mathrm{of}\;\mathrm{tyrosinase}\;=\frac{\left(A-B\right)-\left(C-D\right)}{\left(A-B\right)}\times 100\%$$

where A is the absorbance of the reaction mixture of the enzyme but without test samples, B is the absorbance of the buffer solution without test samples and enzyme, C is the absorbance of the test samples and enzyme, and D is the absorbance of the test sample but without enzyme.

### 3.8. Statistical Analysis

The results were expressed as mean ± standard deviation (SD), and were repeated three to five times. The inhibitory concentration (IC_50_) was calculated by log-Probit analysis.

## Figures and Tables

**Table 1:** Traditional uses and ethnobotanical data of the Peruvian plants used in the current study.

N°	Scientific Name	Common Name ^a^ [9,10,11,12,13,14,15,16,17,29,30,31] ^b^	Family Name	Traditional Uses and Ethnopharmacological Activity [9,10,11,12,13,14,15,16,17,29,30,31] ^b^
1	*Adiantum* cf. *poiretii* Wikstr.	Culantrillo (S)	PTERIDACEAE	Excessive menstrual bleeding, menstrual cramps and vaginal inflammation
2	*Alchornea castaneifolia* (Humb. & Bonpl. ex Willd.) A. Juss.	Iporuro (S)	EUPHORBIACEAE	Rheumatism, arthritis, ulcer, gastritis and muscular pains
3	*Anacardium occidentale* L.	Casho (S), Marañón (S), Castaña de cajú (S), Pepa de la selva (S)	ANACARDIACEAE	Stomach discomfort, antidiarrheal and used as food
4	*Annona muricata* L.	Hojas de Graviola (S), Soursop (E)	ANNONACEAE	For coughs, asthma, and hypertension. Used as antibacterial, antifungal, antioxidant, and anti-inflammatory agents
5	*Baccharis genistelloides* (Lam.) Pers.	Carqueja (S), Cuchu Cuchu (Q), Tres esquinas (S), kimsacucho (Q), Carceja (S), Cadillo (S)	ASTERACEAE	Diabetes/cholesterol; used as an anti-inflammatory agent (liver, kidneys, biliar) and in intestinal disorders
6	*Buddleja americana* L.	Flor Blanca (S)	SCROPHULARIACEAE	Inflammation of womb, ovarian cysts and uterus
7	*Caiophora* cf. *cirsiifolia* C. Presl	Ortiga colorada (S), Ckora-quisa (Q), Pucahitana (Q), Puca-lalay (Q), Puca-Sasay (Q), Pucasique (Q)	LOASACEAE	Used as an antitussive, expectorant, and antipyretic to relieve cold, flu and bronchitis
8	*Capsicum baccatum*	Aji Amarillo (S)	SOLANACEAE	Rheumatism, arthritis, treatment of problems with skin and wounds
9	*Cheilanthes pilosa* Goldm.	Cuti Cuti (Q)	PTERIDACEAE	Diabetes and liver
10	*Chenopodium pallidicaule*	Cañihua (Q)	AMARANTHACEAE	Used in food, such as bread, and for drinks on long trips
11	*Chrysopogon zizanioides* (L.) Roberty	Pachuli (Q)	POACEAE	Depression, insomnia, anxiety, stress, tension, nervousness, inflamed skin and wounds
12	*Chuquiraga spinosa* Less.	Huamanpinta (Q), Care Sirve (Q), Pucacasha (Q), Chuquiraga (Q)	ASTERACEAE	Treatment of kidney disorders; used as an anti-inflammatory (renal), and for gonorrhea, as well as bladder and prostate problems
13	*Clinopodium brevicalyx* (Epling) Harley & A. Granda	Inka muña (Q)	LAMIACEAE	Treatment of diarrhea, gastritis and colic. Antitussive to relieve cold and flu
14	*Clinopodium pulchellum* (Kunth) Govaerts	Panisara (S)	LAMIACEAE	Spiritual cleansing
15	*Cordia Lutea* Lam.	Flor de Overo (S), Overo (S), Overal (S)	BORAGINACEAE	Used as an anti-inflammatory (liver, kidney, bladder, ovaries), and for hepatitis
16	*Cymbopogon citratus* (DC.) Stapf.	Hierba Luisa (S)	POACEAE	Cold, cough, flu and cancer
17	*Desmodium molliculum* (Kunth) DC.	Manayupa (Q), Pie de perro (S), Pata de perro (S), Chancas de comida (S)	FABACEAE	Used in gastritis, wound cleansing; as an anti-inflammatory (kidneys, ovaries) and in wound healing and diarrhea
18	*Dianthus caryophyllus* L.	Claveles (S), Clavelina (S), Clavel de la costa (S)	CARYOPHYLLACEAE	Insomnia, nerves and heart
19	cf. *Endlicheria*	Spingo (S)	LAURACEAE	No reports
20	*Equisetum giganteum* L.	Cola de caballo (S), Shawinco (Q)	EQUISETACEAE	Treatment of prostatitis, used as an anti-inflammatory (bladder, and renal calculous) and to cicatrize infectious injuries and wounds. Also used in cancer
21	*Eucalyptus globolus* L.	Eucalipto (S), Alcanfor Serrano (S)	MYRTACEAE	Antitussive, descongestant, analgesic and anti-spasmodic to relieve cold, cough, bronchitis, flu, asthma and rheuma
22	*Flaveria bidentis* (L.) Kuntze	Mata gusano (S)	ASTERACEAE	Treatment of prostatitis; used as an anti-inflammatory (bladder, renal calculous) and to cicatrize infectious injuries. Used in wounds, cancer, and for cough and bronchitis
23	*Gentianella tristicha* (Gilg) J.S. Pringle	Hercampure (Q)	GENTIANACEAE	Diabetes, diuretic and cholesterol
24	*Gnaphalium dombeyanum* DC.	Arnica (Q), Shymaicho (Q)	ASTERACEAE	Treatment of indigestion, also used as an anti-inflammatory and to cicatrize injuries and skin ulcers
25	*Huperzia crassa* (Humb. & Bonpl. ex Willd.) Rothm.	Trensilla o enredadera (S)	LYCOPODIACEAE	Spiritual cleansing
26	*Hypericum laricifolium* Juss.	Hierba de la fortuna (S), Solitario (S), Chinchango (Q), Abrecaminos (S), Romerillo (S)	CLUSIACEAAE	Luck in love, good fortune, good health, and paludism
27	*Jatropha curcas* L.	Piñones (S), Piñol (S)	EUPHORBIACEAE	Depurative-emetic, wound disinfectant, vaginal infection and sedative
28	*Jatropha macrantha* Müll. Arg	Male Huanarpo (E), Huanarpo macho (S)	EUPHORBIACEAE	Fertility, sexual potency, male impotence and tension
29	*Lupinus mutabilis*	Tarwi (Q)	FABACEAE	Used as food
30	*Matricaria recutita* L.	Labanda (S), Manzanillon (S)	ASTERACEAE	Infections of wounds, vaginal cleansing; used for blood purification, stomach pain, cold and flu. Also used as a laxative, for digestion, and as a sedative
31	*Malesherbia splendens* Ricardi	Veronica (S)	PASSIFLORACEAE	Bronchitis
32	*Ocimum basilicum* L.	Albahaca de olor (S)	LAMIACEAE	For better sleep, headaches and nerves
33	*Otholobium mexicanum* (L. f.) J.W. Grimes	Culen negro (S)	FABACEAE	Diarrhea and cold of the stomach
34	*Otholobium pubescens* (Poir.) J.W. Grimes	Culen Blanco (S)	FABACEAE	Diabetes, colic, constipation, indigestion, laxative and stomach purification
35	*Oreobolus obtusangulus Gaudich./Eleocharis albibracteata* Nees & Meyen ex Kunth	Hierba del caballero (S)	CYPERACEAE	Spiritual cleansing
36	*Peumus boldus* Molina	Boldo (S)	MONIMIACEAE	Anti-inflammatory (liver and kidney)
37	*Phoradendron* sp.	Suelda con suelda (S), Tullma tullma (Q)	LORANTHACEAE	Spiritual cleansing
38	*Phyllanthus niruri* L.	Chanca Piedra (S)	EUPHORBIACEAE	Cleansing (of the stomach, blood); anti-inflammatory (liver, kidneys, gallbladder)
39	*Muehlenbeckia vulcanica* Meisn.	Mullaka (Q), Viruta (S)	POLYGONACEAE	Treatment of diarrhea, bronchitis, asthma, pain, flu, throat and infections
40	*Piper aduncum* L.	Matico (S)	PIPERACEAE	Anti-inflammatory, and treatment of diarrhea
41	*Puya* sp.	Hierba del Carnero (S), Lana de carnero (S), Hierba de Borrego (S), Solitario (S), Abrecaminos (S)	BROMELIACEAE	Tumors and infections
42	*Rosmarinus officinalis*	Romero (S)	LAMIACEAE	Treatment of liver and bladder disorders, anti-inflammatory to relieve rheumatism and peripheral vascular diseases
43	*Salvia hispanica* L.	Chia (S)	LAMIACEAE	Used as food
44	*Sambucus peruviana* H. B. K.	Sauco (S), Tilo (S), Saucotillo (S)	CAPRIFOLIAEAE	Bronchitis, yellow fever, inflammation of the kidneys and cough
45	*Senna* sp.	Hojas de sen (S)	FABACEAE	Purgative, constipation, and cleansing of the stomach
46	*Smallanthus sonchifolius* (Poepp.) H. Rob.	Hojas de Yacon (S), Yacon (S), Llacon(Q)	ASTERACEAE	Diabetes, cholesterol, kidney and inflammation of the prostate
47	*Taraxacum officinale* F.H. Wigg.	Diente de leon (S), Amargon (S), Lengua de Leon (S)	ASTERACEAE	Liver, stomach, inflammation, (ovaries). Used for depurative and diuretic effects
48	*Tiquilia Paronychioides* (Phil.) Rich.	Flor de arena (S)	BORAGINACEAE	Anti-inflammatory (ovaries, kidneys) and used in urinary infections
49	*Valeriana* sp.	Raiz de valeriana (S)	CAPRIFOLIACEAE	Treatment of sleep disorders and sedative properties
50	*Werneria nubigena* Kunth	Condor (S)	ASTERACEAE	Calmative effects

^a^ Spanish (S), Quechua (Q), English (E); ^b^ Based on ethnobotanical survey.

**Table 2:** Tyrosinase inhibitory activity of 70% methanol extracts of Peruvian medicinal plants.

No	Scientific Name	Voucher Specimen	Part Used	Yield (%)	Inhibition % (500 μg/mL)
1	*A.*cf. *poiretii* Wikstr.	A9	Ar	14.1	<1 ± 1.9
2	*A. castaneifolia* (Humb. & Bonpl. ex Willd.) A. Juss.	P19	Lv	16.1	<1 ± 0.8
3	*A. occidentale* L.	A41	Fr	25.6	42.51 ± 7.2
4	*A. muricata* L.	A5	Lv	19.5	<1 ± 2.9
5	*B. genistelloides* (Lam.) Pers.	P78	Ar	19.7	18.43 ± 2.5
6	*B. americana* L.	A19	F	8.5	8.09 ± 0.7
7	*C.* cf. *cirsiifolia* C. Presl	A25	Ar	7.0	22.54 ± 3.4
8	*C. baccatum*	A46	Fr	50.9	8.91 ± 1.1
9	*Ch. pilosa* Goldm	P17	Ar	14.3	40.35 ± 2.0
10	*Ch. pallidicaule*	A50	S	1.1	2.83 ± 3.4
11	*Ch. zizanioides* (L.) Roberty	A13	Lv	9.1	4.99 ± 1.8
12	*Ch. spinosa* Less	P77	Ar	28.7	<1 ± 2.6
13	*Cl. brevicalyx* (Epling) Harley & A. Granda	P11	Lv	26.8	<1 ± 2.0
14	*Cl. pulchellum* (Kunth) Govaerts	A15	Lv	4.1	12.55 ± 0.9
15	*C. lutea* Lam	P79	F	12.3	10.6 ± 3.1
16	*C. citratus* (DC.) Stapf	A18	Lv.	6.8	29.82 ± 0.9
17	*D. molliculum* (Kunth) DC.	P80	Lv	23.8	20.65 ± 2.9
18	*D. caryophyllus* L.	A20	F	2.5	30.36 ± 3.4
19	Cf. *endlicheria*	A43	S	10.1	6.88 ±0.7
20	*E. giganteum* L.	P55	Ar	11.2	<1 ± 5.2
21	*E. globolus* L.	P39	Lv	18.0	10.93 ± 1.9
22	*F. bidentis* (L.) Kuntze	A31	Lv	7.9	<1 ± 2.4
23	*G. tristicha* (Gilg) J.S. Pringle	P7	Ar	30.3	22.54 ± 1.3
24	*G. dombeyanum* DC.	A29	Lv	3.7	5.67 ± 4.0
25	*H. crassa* (Humb. & Bonpl. ex Willd.) Rothm.	A27	Lv.	12.3	<1 ± 0.3
26	*H. laricifolium* Juss.	A10	Lv	15.9	74.00 ± 2.1
27	*J. curcas* L.	A39	S	1.9	<1 ± 2.9
28	*J. macrantha* Müll. Arg.	P2	R	23.6	37.11 ± 5.7
29	*L. mutabilis*	A47	S	10.2	<1 ± 3.6
30	*M. recutita* L.	A12	Lv	7.9	40.49 ± 2.1
31	*M. splendens* Ricardi	A22	Lv	7.7	38.06 ± 1.8
32	*O. basilicum* L.	A11	Lv	5.6	28.10 ± 4.1
33	*O. mexicanum* (L. f.) J.W. Grimes	A3	Ar	6.1	19.57 ± 2.1
34	*O. pubescens* (Poir.) J.W. Grimes	A4	Ar	19.6	31.58 ± 0.8
35	*O. obtusangulus Gaudich /E. albibracteata* Nees & Meyen ex Kunth	A28	Lv	3.9	17.41 ± 1.1
36	*P. boldus* Molina	P40	Lv	32.5	<1 ± 2.6
37	*Phoradendron* sp.	P83	Lv	57.1	<1 ± 1.2
38	*P. niruri* L.	P5	Lv	12.9	11.34 ± 6.3
39	*M. vulcanica* Meisn	P82	Lv	20.1	57.1 ± 3.0
40	*P. aduncum* L.	P44	Lv	13.4	<1 ± 1.1
41	*Puya* sp.	A38	Ar	1.9	12.55 ± 10.3
42	*R. officinalis*	P81	Lv	20.4	8.22 ± 3.1
43	*S. hispanica* L.	P4	S	3.5	44.31 ± 3.4
44	*S. peruviana* H. B. K.	A24	Lv	8.1	18.49 ± 2.4
45	*Senna* sp.	A7	Lv	8.1	44.13 ± 2.3
46	*S. sonchifolius (Poepp.)* H. Rob.	A6	Lv	5.3	<1 ± 1.1
47	*T. officinale* F. H. Wigg	P49	Ar + F	4.8	60.8 ± 4.1
48	*T. paronychioides* (Phil.) Rich.	P36	Ar	18.5	10.93 ±1.3
49	*Valeriana* sp.	P42	R	52.7	17.04 ± 3.7
50	*W. nubigena* Kunth	A33	Lv	8.5	1.21 ± 1.5
**Arbutin**					92.3 ± 2.2

Data represent the mean ± standard error media of the evaluated parameter. Ar = Arial part, F = Flowers, Fr = Fruits, Lv = Leaves, R = Root, S = Seed.

**Table 3:** IC_50_ values of tyrosinase inhibitory from various solvent extracts of *H. laricifolium* Juss.

Extracts	Concentration (μg/mL)	Inhibition (%)	IC_50_ (μg/mL)
*H. laricifolium* Juss. Methylene chloride	1000	<1 ± 4.3	-
*H. laricifolium* Juss. 70% MeoH	1000	80.9 ± 1.1	122.1± 4.1
	500	77.7 ± 2.8	
	100	46.1 ± 2.5	
*H. laricifolium* Juss. 50% MeoH	1000	22.3 ± 5.4	-
Arbutin	500	98.2 ± 0.9	42.0 ± 0.8
	50	63.8 ± 0.1	
	10	13.7 ± 1.8	

**Table 4:** Percentage of tyrosinase inhibition and amounts of compounds from *H. laricifolium* Juss. and positive control: arbutin and kojic acid. The concentration of the compounds and positive control was 100 μg/mL.

Compounds	Inhibition (%)	IC_50_ (μM)	Regression Equation	Correlation Coefficient (R^2^)	Active Compounds (μg)
Protocatechuic acid (**1**)	NI	-	Y = 0.6294x + 2.0882	0.9829	8.4
*p*-Hydroxybenzoic acid (**2**)	8.3 ± 4.03	-	Y = 0.1963x + 17.276	0.9479	5.62
Chlorogenic acid (**3**)	NI	-	Y = 0.3199x + 10.849	0.9889	17
Vanilic acid (**4**)	NI	-	Y = 0.1824x + 13.326	0.9198	5.07
Caffeic acid (**5**)	NI	-	Y = 0.1641x + 1.1179	0.9994	2.39
Kaempferol 3-*O*-glucuronide (**6**)	NI	-	Y = 0.0948x + 9.0847	0.9407	3787.9
Quercetin (**7**)	99.7 ± 0.28	14.29 ± 0.3	Y = 0.1392x + 35.232	0.9018	720.5
Kaempferol (**8**)	30 ± 1.97	-	Y = 0.2578x + 2.0698	0.9992	0.82
Arbutin	86.01 ± 1.6	110.4 ± 1.9	-		
Kojic acid	99.8 ± 0.5	8.0 ± 0.5			
